# Effect of different solutions in reversing the damage caused by radiotherapy in dentin structure

**DOI:** 10.4317/medoral.23499

**Published:** 2020-05-10

**Authors:** Fabiane Lopes, Manoel Sousa-Neto, Anna Akkus, Ricardo Silva, Alexandra de Queiroz, Harley de Oliveira, Renato Roperto

**Affiliations:** 1 DDS, MS, PhD. Department of Restorative Dentistry, School of Dentistry of Ribeirão Preto, University of São Paulo, SP, Brazil; 2 MS. Department of Macromolecular Science and Engineering, School of Engineering, Case Western Reserve University, Cleveland, OH, USA; 3 DDS, MS, PhD. Department of Pediatric Dentistry, School of Dentistry of Ribeirão Preto, University of São Paulo, SP, Brazil; 4 MD, PhD. Department of Internal Medicine, Faculty of Medicine of Ribeirão Preto, University of São Paulo, SP, Brazil; 5 DDS, MS, PhD. Department of Comprehensive Care, School of Dental Medicine, Case Western Reserve University, Cleveland, OH, USA

## Abstract

**Background:**

Previous studies have shown that radiotherapy of the head and neck region can cause direct changes in dental structure. This study evaluated the effect of different solutions on the dentin chemical composition and collagen structure of irradiated dentin.

**Material and Methods:**

Sixty maxillary canines were distributed in 2 groups (n=30): non-irradiated and irradiated (radiotherapy: X-rays of 6 MV in 30 cycles of 2 Gy to 60 Gy). The teeth were sectioned, sanded, and polished to obtain 3x3x2 mm fragments, which were redistributed in 3 subgroups (n=10) according to the treatment employed: chlorhexidine 2% (CL), chitosan 0.2% (QT), and 0.5 M carbodiimide (EDC). The samples were analyzed in FTIR at time zero (T0-control) and after 1 (T1), 3 (T3), and 5 (T5) minutes of immersion in the tested solutions. The data for the areas of the carbonate (C), amide I (AI) bands, and the ratio between the areas of the amide III/proline and hydroxyproline (AIII/PH) bands were analyzed using ANOVA and Tukey test (α=5%).

**Results:**

QT showed lower C values at T1, T3, and T5 (*P*<0.0001), presenting lower values when compared to CL and EDC subgroups (*P*<0.05). AI values at T3 and T5 were higher than T0-control and T1, independently of the radiotherapy and dentin treatment factors (*P*<0.05). At T0-control, the AIII/PH ratio was lower in the irradiated group (*P*<0.05), whereas the EDC treatment at T1, T3, and T5 and QT at T3 and T5 increased these values (*P*<0.05), making them similar to non-irradiated subgroups (*P*>0.05).

**Conclusions:**

Radiotherapy changes the secondary structure of collagen, and EDC was able to restore collagen integrity after 1 minute of immersion, without changing dentin inorganic composition.

** Key words:**Radiotherapy, collagen, dentin, FTIR, chemical composition.

## Introduction

Radiotherapy is used for treatment of head and neck cancer and can be used as primary therapy, as adjunctive to surgical treatment and chemotherapy, or as palliative treatment in the final and inoperable stages of the disease, the treatment mode being determined by tumor type, stage, and locality (1,2).

Radiation therapy consists of high energy ionizing radiation that interacts with the cells generating electrons that ionize the medium and lead to the rupture of the deoxyribonucleic acid (DNA) chains and water hydrolysis, promoting the inhibition of cell division in addition to the production of free radicals that result in the destruction of neoplastic tissues (3). Although the radiotherapy treatment is performed in the most conservative manner, healthy surrounding tissues such as bone, mucosa, teeth, and salivary glands can suffer damage in the process (4).

Previous studies have shown that radiotherapy of the head and neck region can cause direct changes in dental structure (5), such as changes in the dentin chemical composition (6), besides changes in the peptide chains, dehydration of the collagen fibers (7), and increased expression of metalloproteinases (MMPS) (8). These alterations may interfere with tooth-restoration interaction, reducing the bond strength of adhesive systems (9) and consequently increasing the susceptibility to failure of the restorations (10).

In parallel, studies have shown that the longevity of the restorations may be related to the previous treatment of the dentin, which can contribute to the maintenance of the stability of the hybrid layer and consequently increase the longevity of the adhesive interface (10). Among the solutions that have been used for this purpose, chlorhexidine digluconate is a biocompatible antimicrobial solution that has the ability to inhibit the MMPs, enzymes that degrade the collagen fibrils, contributing to the maintenance of the long term bond strength values (11). Carbodiimide (1-Ethyl-3- [3-dimethylaminopropyl]) (EDC) is a nontoxic substance that leaves no residue after treatment and which has the ability to stimulate the formation of crosslinks, improving the mechanical properties of dentin collagen while inhibiting MMPs (12), thereby preventing the degradation of adhesive interfaces (13). Similarly, researchers have tried to improve the biological and mechanical properties of collagen with a reinforcing matrix through the use of chitosan, which is a hydrophilic biopolymer with a large number of free hydroxyl and amino groups, non-toxic, antifungal, antioxidant, anti-inflammatory, healing, biocompatible and biodegradable, derived from the deacetylation of chitosan obtained from crustaceans’ shells (14).

The study of the chemical changes of the dentin surface after different treatments has been performed using Fourier transform infrared spectroscopy (FTIR) (15). FTIR allows the identification and characterization of the molecular structures of the organic and inorganic compounds of the dentin, non-destructively, through the absorption spectrum of the sample that is generated from the vibrational modes of the molecules, creating parameters of comparison between the analyzed areas (15).

The increase in technological innovation and the improvements in cancer treatment in the last decades have resulted in an increase in the survival rate and an improvement in the quality of life of the patients affected (16), albeit radiotherapy treatment performed in the head and neck region generates side effects in the oral cavity, such as the rapid progression caries (17), leading to the need for restorative treatments which in turn are hampered due to the damage of the dental structure (1,9,10). Thus, clinical protocols for treatment of irradiated teeth should be studied in order to minimize the effects of radiotherapy on the dentin chemical composition and dentin collagen structure, which directly interfere in the formation of the restorations’ adhesive interface. Thus, the objective of the present study was to evaluate the effect of radiotherapy on collagen structure and dentin chemical composition, as well as the ability of chorhexidine, chitosan, and EDC to minimize this effect.

Materials and Methods

Sample size calculation was determined using F-tests and ANOVA statistical tests for fixed, special, main effects and interactions using G*Power software, version 3.1.9.2 (Heinrich Heine University, Düsseldorf, Germany). The fixed parameters were error type α=0.05, statistical power β=0.8, numerator dF=2 and number of groups = 6. Based on previous studies, the effect size was set to 0.45 (18). The minimum estimated sample size was 8.5 specimens per group. A sample size of 10 specimens (n=10) was chosen for each group.

Sixty maxillary canines, with absence of caries lesions and structural anomalies were obtained from the University Human Teeth Biobank, washed in running water for 24 hours, and had their external surface cleaned by ultrasonic scraping. The teeth were organized in eppendorf tubes containing artificial saliva (pH 7), renewed daily, and stored (37ºC, 100% humidity). The samples were randomly assigned to 2 groups (n=30) according to the radiotherapy regimen (non-irradiated - control, and irradiated), and 3 subgroups (n=10) according to the solution used for dentin treatment (chlorhexidine, carbodiimide - EDC, and chitosan).

The thirty teeth assigned to receive radiation were placed in a plastic support with the long axis perpendicular to the ground. The plastic support was aligned equidistant to the center of the beam to ensure uniform dose rate (400 UM/min). In order to keep the teeth in a humid environment, simulating the characteristics of the oral cavity, the plastic support was filled with distilled water, completely covering all the teeth. Treatment was delivered by a computer-assisted linear accelerator using 6 MV X-rays (RS 2000, RAD Source Technologies, Inc., Suwanee, GA, USA). A cumulative radiation dose of 60 Gy fractionated in 30 fractions (2 Gy per fraction) was delivered in 5 consecutive days per week, over 6 weeks. Between the irradiation cycles, the teeth were stored in artificial saliva (37ºC, 100% humidity). Also, the samples assigned to the non-irradiated group had the artificial saliva renewed daily and stored in the same conditions.

- Specimens preparation

The teeth were placed in acrylic resin plates and fixed with hot glue. The plates were individually mounted on the Isomet 1000 low speed saw (Buehler, Lake Forest, IL, USA) and sectioned with a diamond blade of 0.5 mm thickness (South Bay Technology, San Clement, CA, USA) at a constant speed of 300 rpm, under refrigeration, in order to obtain intraradicular cervical dentin block. Then, the fragments were flattened and polished with #1200 grit SiC paper on a rotary polishing machine (APL-4, Arotec S/A Ind. e Comércio, São Paulo, SP, Brazil), under abundant cooling, so that the surfaces became as parallel as possible. The final dimension of the samples was 3 x 3 x 2 mm.

The samples obtained were submitted to an ultrasonic bath for 10 minutes, and the surface was treated with 37% phosphoric acid (Ultradent Products, South Jordan, UT, USA) for 15 seconds and rinsed for 15 seconds. The specimens were individually stored in numbered eppendorf tubes containing distilled water at 4ºC.

- Fourier-transform infrared spectroscopy (FTIR) analysis

The analysis of the dentin chemical composition and dentin collagen structure of all sixty samples was performed using the Fourier Transform Infrared Spectrometer (Agilent 630FTIR Spectrometer, Agilent Technologies, Santa Clara, CA, USA) with a DTGS detector using a diamond attenuated reflection accessory (ATR) sample press. To ensure that the FTIR analyses were always done at the same point, a high-speed spherical drill was used to mark the surface of the specimens.

Initially, the system was calibrated according to the manufacturer's instructions and the samples were positioned with the face to be analyzed facing the crystal and held in position by the press. Three FTIR spectra of each sample were obtained with 0.5 cm-1 resolution, with 50 scans in the range of 4000–650 cm-1, and were recorded using the Microlab PC Software. The infrared bands considered for this study were: 850 cm-1 (carbonate), 960 cm-1 (phosphate), 1630 cm-1 (amide I), 1240 cm-1 (amide III), and 1450 cm-1 (proline and hydroxyproline). The bands were normalized by the phosphate band, and the areas of the other bands were calculated using the Microcal Origin 6 (Microcal Origin Software, MA 01060, Northampton, USA) for comparison between groups. To evaluate the integrity of the collagen triple helix, the peak absorbance ratios of 1240 cm-1/1450 cm-1 were considered (15,19).

The analyses were performed in all the samples at four different moments: T0 - control, without immersion in any solution; T1 - after one minute of immersion; T3 - after 3 minutes of immersion; and T5 - after 5 minutes of immersion, wherein in each moment the analysis was repeated 3 times in the same point, and the average of the calculated areas was considered.

- Dentin Treatments

Ten samples (n=10) from each group (non-irradiated and irradiated) were randomly submitted to the treatment with one of the following solutions: 2% chlorhexidine digluconate solution; 0.2% chitosan acetic acid solution (Acros Organics, Geel, Belgium); or 0.5 M aqueous solution of carbodiimide (1-ethyl-3- (3-dimethylaminopropyl) carbodiimide hydrochloride - EDC (ProteoChem, Denver, USA).

The specimens were immersed on the test solution and then submerged in distilled water for 30 seconds to remove the irrigation solutions. The specimens were gently dried on absorbent paper and immediately evaluated by FTIR. This procedure was repeated to obtain data of 1 (T1), 3 (T3), and 5 (T5) minutes of immersion. The solutions were renewed before each immersion period to simulate clinical conditions and to prevent saturation. All test procedures were performed at room temperature (25°C).

- Statistical Analysis

The data of the band area related to carbonate, amide I, and the ratio between the areas of amide III / proline and hydroxyproline peaks (integrity of the collagen triple helix) were examined for normal distribution (Shapiro–Wilk test, *P*>0.05) and homogeneity of variance (Levene’s test, *P*>0.05). The data were analyzed by two-way ANOVA in a split-plot arrangement with the plot represented by radiotherapy (non-irradiated and irradiated) and dentin treatment (chlorhexidine, carbodiimide – EDC, and chitosan); and the subplot represented by the application time (T0 – control, T1 – 1 minute, T3 – 3 minutes, and T5 – 5 minutes). Pairwise multiple comparison procedures were performed using Tukey’s test. All tests were performed using the Sigmaplot 11.0 software package (Systat Software Inc., San Jose, CA, USA), with the significance level set at α=0.05.

## Results

- Evaluation of the carbonate band areas 

ANOVA results for carbonate band areas showed statistical differences between dentin treatments (*P*<0.0001) and between application times (*P*<0.0001), as well as in the interactions between dentin treatment and application time (*P*<0.0001); and no differences between radiotherapy factors (non-irradiated and irradiated).

For chlorhexidine and EDC there was no difference between the application times (*P*>0.05), however chitosan reduced the carbonate bands area at T1 (*P*<0.0001), which presented intermediate values, while T3 and T5 showed the lowest values (*P*<0.0001; Table 1). Also, when comparing the treatments, chitosan had lower carbonate values then chlorhexidine and EDC at T1, T3, and T5 (*P*<0.05; Table 1).

- Evaluation of the amide I band areas 

ANOVA results for amide band areas showed statistical differences between application times (*P*<0.0001), independent of the radiotherapy and dentin treatment factors, wherein T3 and T5 had higher amide I values than T0 (control) and T1 (*P*<0.05; Table 2).

- Evaluation of the ratio between the areas of amide III / proline and hydroxyproline peaks 

ANOVA results for the ratio values between the areas bands of amide III / proline and hydroxyproline showed statistical differences between radiotherapy factors (*P*<0.0001), between dentin treatments (*P*=0.0377), and in the interactions between radiotherapy and dentin treatment (*P*<0.0001), radiotherapy and application times (*P*<0.0001), and between the three factors, radiotherapy, dentin treatments, and application times (*P*<0.0001).

At T0 (control), the amide III / proline and hydroxyproline ratio was reduced by the radiotherapy protocol (*P*<0.05). However, the application of EDC at T1, T3, and T5 as well as the application of chitosan in T3 and T5, increased the amide III / proline and hydroxyploline ratio in irradiated teeth (*P*<0.05), wherein these values were similar to non-irradiated teeth (*P*>0.05). The application of chlorhexidine to non-irradiated teeth did not alter the amide III / proline and hydroxyproline ratios. However, the application of chlorhexidine in irradiated teeth increased the amide III / proline and hydroxyproline ratio in T5 when compared to T0 (control) and T1 (*P*>0.05), and in T3 presented intermediate values, sometimes similar to T0 (control) and T1, or similar to T5; however, T1, T3, and T5 values were significantly different between non-irradiated and irradiated teeth for the same treatment and application time (Table 3).

**Table 1:**
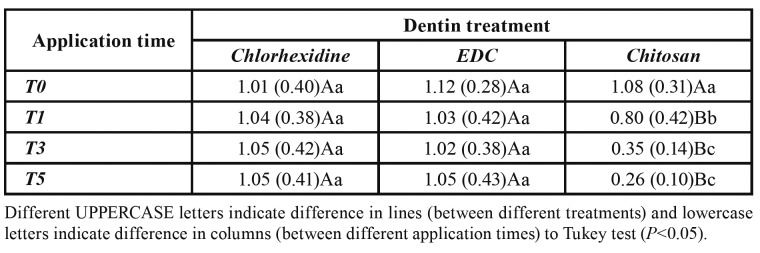
Mean (standard deviation) of the area of the carbonate bands for the different treatments in the different experimental times.

**Table 2:**
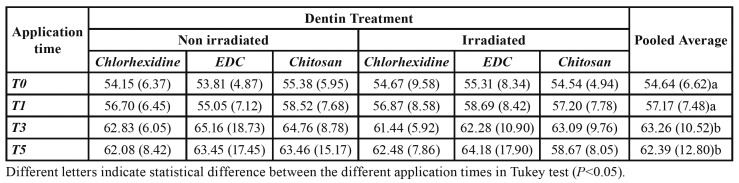
Mean (standard deviation) of the area of the amide I bands for the different treatments at the different experimental times.

**Table 3:**
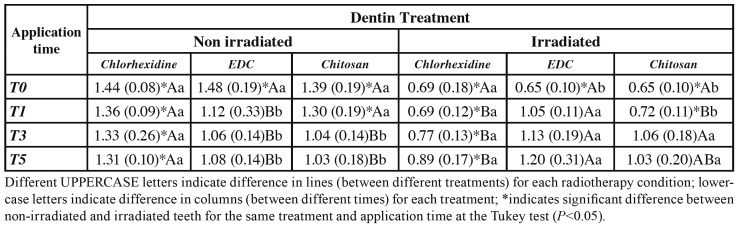
Mean (standard deviation) of the ratio between the area of the amide III / proline and hydroxyproline bands (collagen triple helix integrity) for the different dentin treatments in the different application times.

## Discussion

The dentin tissue is composed of approximately 70% by weight inorganic content, 20% organic content and 10% water (20). The inorganic phase of dentin consists of carbonate and phosphate, which form the hydroxyapatite, whereas the organic phase consists of 90% in type I collagen fibers and 10% of non-collagenous matrix proteins, wherein these two phases are strongly bound by the water molecules (20). In the present study, FTIR analysis was performed to identify changes in the chemical composition of dentin and collagen structure through the evaluation of carbonate, amide I, amide III, and proline and hydroxyproline bands.

Considering the FTIR analysis, the amide band I (1630 cm-1) is one of the main absorption bands of the collagen peptide group, and represents the stretches between C=O, with little contribution of N-H deformation, and thus, the decrease in the area of the amide band I is related to the damage to the primary structure of the collagen fibers (21). Moreover, for type I collagen fibrils, the integrity of its secondary structure can be verified when the ratio of amide III to proline and hydroxyproline (1450 cm-1) is greater than or equal to 1 (15), once amide III (1240 cm-1) is related to the C-N and N-H stretch (15) and that less amount or lack of hydroxyproline causes the collagen to lose the triple helix conformation. Furthermore, the carbonate area bands (850 cm-1) were used to evaluate the inorganic composition in dentin.

Thus, considering the results of the present study, it can be affirmed that the radiotherapy caused direct damage to the collagen secondary structure, specifically, in the connections between the peptide chains (22), since it caused the loss of the triple helix conformation, while causing no changes in the primary structure of the collagen nor in its inorganic portion. Radiotherapy X-rays act in the presence of water through the formation of free radicals of hydrogen and hydrogen peroxide (23), capable of acting as a strong oxidant, causing denaturation and fragmentation of the dentin collagen fibers network (24), besides activating metalloproteinases (8). This alteration of the collagen secondary structure may explain changes in its physical and mechanical properties observed in other studies, such as increased solubility (25), decrease in microhardness (7) and tensile strength (1), besides compromising the integrity of the hybrid layer (2,7) and the bond strength of the adhesive interfaces (7,9).

Although the chlorhexidine is able to preserve the hybrid layer, maintaining the structure and stabilizing the collagen fiber network (11), in the present study this solution was not able to restore the integrity of the triple collagen helix in the irradiated teeth, since even after 5 minutes of immersion (T5) the irradiated teeth continued with the ratio amide III / proline and hydroxyapoline similar to the control (T0). The amide I values increased after 3 and 5 minutes of immersion, both in the irradiated group and in the non-irradiated group, which is probably related to the action of chlorhexidine, which inhibits the action of MMPs, through electrostatic attachment to collagen (11), helping to maintain its primary structure. However, its use after damage to secondary collagen structure has already been caused by radiation therapy does not reverse the damage. Soares, *et al*. [2011] (2), found that the use of mouthwashes with chlorhexidine 0.12%, during the radiotherapy treatment, only partially restored the damages to the dentin mechanical properties.

In the present study, chitosan progressively reduced the carbonate values from the first minute of immersion, indicating a loss of the inorganic phase of the dentin, which can be attributed to its chelating characteristic (14,26). Simultaneously with chitosan’s demineralizing effect on the dentin surface, it has the ability to bind with collagen fibrils by means of electrostatic attraction, involving them, which increases the collagen resistance (27). According to Persadmehr *et al*. [2014] (27), the affinity between chitosan and collagenase causes a protection of collagen fibrils by blocking access to MMPs and hampering their actions. Thus, in the present study, chitosan restored the integrity of the irradiated dentin collagen after 3 (T3) and 5 (T5) minutes of immersion, and, similarly to chlorhexidine, also increased amide I values in irradiated and non-irradiated groups.

Regarding EDC dentin treatment, the FTIR analysis verified that after 1 minute (T1), the integrity of the triple helix of the collagen of the irradiated group was restored, and was maintained after 3 (T3) and 5 (T5) minutes of treatment. EDC is able to aggregate amino acids into peptides, being a non-specific cross-linker of unique ability to activate the carboxyl group of the glutamic and aspartic acids present in the protein chains (28), which results in cross-links without residual reactive groups (13). Thus, its ability to stimulate the formation of cross-links (13) allowed a rapid restructuring of the secondary structure of the collagen damaged by the radiotherapy. In addition, as well as chlorhexidine and chitosan, its ability to inhibit MMPs (12,29) also resulted in an increase in amide I values in both irradiated and non-irradiated groups.

The present *in vitro* study showed that the radiotherapy alters the secondary structure of collagen. In addition, among the three solutions tested, the use of EDC is a promising alternative for the treatment of dentin in post-head and neck radiotherapy patients, once it was able to restore collagen integrity after 1 minute of immersion, without changing the inorganic composition, which is an advantage over chitosan. Further studies should be conducted to evaluate the advantages of these solutions regarding bond strength and longevity of the adhesive interfaces of irradiated teeth in order to establish an optimal restorative protocol for these patients.
